# Look Up for Healing: Embodiment of the *Heal* Concept in Looking Upward

**DOI:** 10.1371/journal.pone.0132427

**Published:** 2015-07-10

**Authors:** N. D. Leitan, B. Williams, G. Murray

**Affiliations:** Faculty of Health, Arts and Design, Swinburne University of Technology, Melbourne, Australia; University of Udine, ITALY

## Abstract

**Objective:**

Conceptual processing may not be restricted to the mind. The *heal* concept has been metaphorically associated with an “up” bodily posture. Perceptual Symbol Systems (PSS) theory suggests that this association is underpinned by bodily states which occur during learning and become instantiated as the concept. Thus the aim of this study was to examine whether processing related to the *heal* concept is promoted by priming the bodily state of looking upwards.

**Method:**

We used a mixed 2x2 priming paradigm in which 58 participants were asked to evaluate words as either related to the *heal* concept or not after being primed to trigger the concept of looking up versus down (Direction – within subjects). A possible dose-response effect of priming was investigated via allocating participants to two ‘strengths’ of prime, observing an image of someone whose gaze was upward/downward (low strength) and observing an image of someone whose gaze was upward/downward while physically tilting their head upwards or downwards in accord with the image (high strength) (Strength – between subjects).

**Results:**

Participants responded to words related to *heal* faster than words unrelated to *heal* across both “Strength” conditions. There was no evidence that priming was stronger in the high strength condition.

**Conclusion:**

The present study found that, consistent with a PSS view of cognition, the heal concept is embodied in looking upward, which has important implications for cognition, general health, health psychology, health promotion and therapy.

## Introduction

Physical and psychological healing are metaphorically associated with an “up” bodily posture. When someone is physically healed, they can “get up” and play again, and when psychologically healed they can get “up and about” (socialising, etc.; [1, 2]). Embodied cognition suggests that the relationship between up bodily posture and *heal* may not be just metaphorical, but causal. In this study, we examined the relationship between the concept *heal* and physically looking upward, from an embodied cognition perspective.

### Perceptual Symbol Systems theory

Traditional theories of cognition assert that mental representations are amodal symbols (e.g., [3–5]). Such theories construe perceptual and motor content as mere inputs and outputs which are not important for conceptual processing [6]. The contemporary embodied approach to cognition, in contrast, proposes that the body functions as a constituent of the mind, and therefore elevates perceptual content to being intrinsic to, rather than a servant of, cognition [7, 8].

Perceptual Symbol Systems (PSS) theory [9] is one of the most comprehensively articulated and critiqued embodiment theories. PSS theory posits that cognition derives from bodily interactions with the world, which are consequently represented in sensorimotor, proprioceptive, introspective, and emotional areas of the brain. The perceptions, actions, proprioceptions, introspections and emotions which occur during the processing of a concept are termed “embodiments” of that concept [9, 10]. Thus, PSS theory refers to concepts as being “grounded” in their embodiments; that is, concepts exist in the brain as groups of embodiments [11]. For example, Schubert and Koole [12] made participants covertly clench their fist (versus making a neutral hand gesture: index and middle finger sideways like a scissor) and rate themselves on various dimensions of power. The study found that male participants rated themselves as more powerful when making a fist compared to a neutral gesture, thus suggesting that one embodiment of power might be a clenched fist (at least for males) (see also [13]). Other studies have used similar designs, finding that upright posture or looking towards a higher vertical plane might be another embodiment of the *power* concept [6, 14]. Thus together, embodiments such as a clenched fist and upright posture form the *power* concept.

Converging empirical evidence from cognitive/behavioral, neuroimaging, and brain lesion studies supports the idea that the brain’s modality-specific systems are involved in conceptual cognition (for a review see [11]). For example, Wu and Barsalou [15] tested the hypothesis that if people use the brain’s modality-specific systems to process concepts, the manipulation of perceptual variables such as occlusion should affect the process. In order to test this hypothesis they asked half the participants to generate properties for isolated noun concepts (e.g., lawn) and the other half to generate properties for noun concepts which included a modifier (e.g., rolled-up lawn). As predicted, responses to noun concepts which included modifiers revealed more internal properties (e.g., dirt and roots) than isolated noun concepts, suggesting that perceptual variables (from the brain’s modality-specific systems) are utilised in the processing of conceptual knowledge (see also [16–18]).

Kellenbach, Brett, and Patterson [19] conducted a positron emission tomography study in which participants were presented with a property and had to decide whether it could be predicated of each object in the block or not (e.g. was each object in the block “colorful”). Three properties were tested across blocks; colorful vs monochromatic, loud vs silent and small vs large (and a control condition). Results revealed that, relative to the control condition, the modal area relevant to the property of the block became active, suggesting that participants used modality-specific brain areas when assessing the properties of the objects presented in the block. The author’s interpretation was that as each name of an object in the subsequent block was read (e.g. drill), participants conceptualised the object using modality-specific brain areas and assessed whether it contained the property (e.g. “loud” from the auditory area). If the author’s account of their findings is accurate, then predication may utilise modality-specific brain areas (see also [20, 21, 22]).

Under PSS, the simulation process underlies the activation of concepts. Simulation is the modal “reconstruction” of a concept via activation of the embodiments which constitute it. It is proposed to utilise the brain’s modal system, which includes sensorimotor, emotional, introspective and proprioceptive systems, and is implicated in understanding as well as action and perception [23]. When an embodiment is experienced, it triggers simulation of the concept it is a constituent of, including the other embodiments forming the concept, in a process termed “the inference process of pattern completion” [9, 10, 24]. The context in which the embodiment is experienced is crucial since the same embodiment can be a constituent of multiple concepts. For example, the “punch” embodiment can be a part of the *celebration* or *fight* concepts. Simulation of the “punch” embodiment in the context of a victory would be more likely to activate simulation of the *celebration* concept rather than the *fight* concept [24].

### Embodiment of the heal concept in looking upward

Embodiment of the “up” spatial dimension, operationalised in different ways including looking upward, has been well researched and empirical studies have found that it is associated with concepts such as *non-depression* (as opposed to depressed being down; [25]), *happiness* [2], and *positive affect/valence* [26, 27].

Embodied cognition theorists have suggested that *heal* can be embodied in the “up” spatial dimension [2, 28]. This is derived from bodily experiences of being healed where the whole body takes an upright posture or looks upward (head lifted upward). Both are also metaphorically associated with healing as demonstrated linguistically by phrases such as “I’m *up* and about” when feeling better after illness. We chose to examine embodiment of the *heal* concept in looking upward since it encompassed an upright posture and accentuated it.

### Enhancing simulation

PSS theory implies that triggering simulation of an embodiment using two modalities might strengthen simulation of the embodiment, and consequently the concept it is a constituent of. Unlike computational and connectionist theories of cognition [3, 5], which posit transduction (i.e. stripping of perceptual content) of modal states into amodal symbols or nodes which represent or trigger concepts [29, 30], PSS theory implies independent and additive effects of different modal states to represent an embodiment. [9, 23, 31]. Thus, for the former, visual and motor inputs of the same state (e.g., image of someone gazing up (visual) and physically looking up (postural) would be transduced into a *single* amodal symbol of “looking up” which might go on to trigger the concept of *healing*. Whereas in the latter PSS account, the independent visual and motor inputs might have *additive* effects on the strength of “looking up” since *both* inputs contribute to the embodiment, consequently this might allow for a stronger conceptualization of the *heal* concept (a similar idea has been alluded to by [32]). For example, simulation of looking upward triggered via visual perception (e.g., viewing an image of a person looking up) and congruent motor movement (e.g., the posture of tilting the head upwards up) should promote simulation of *heal* to a greater degree than triggering simulation of looking upward via visual perception *or* motor movement *in isolation*. To the best of our knowledge, this was the first study to examine the effect of combing primes on the simulation of a concept.

### The present study

**Fig 1:**
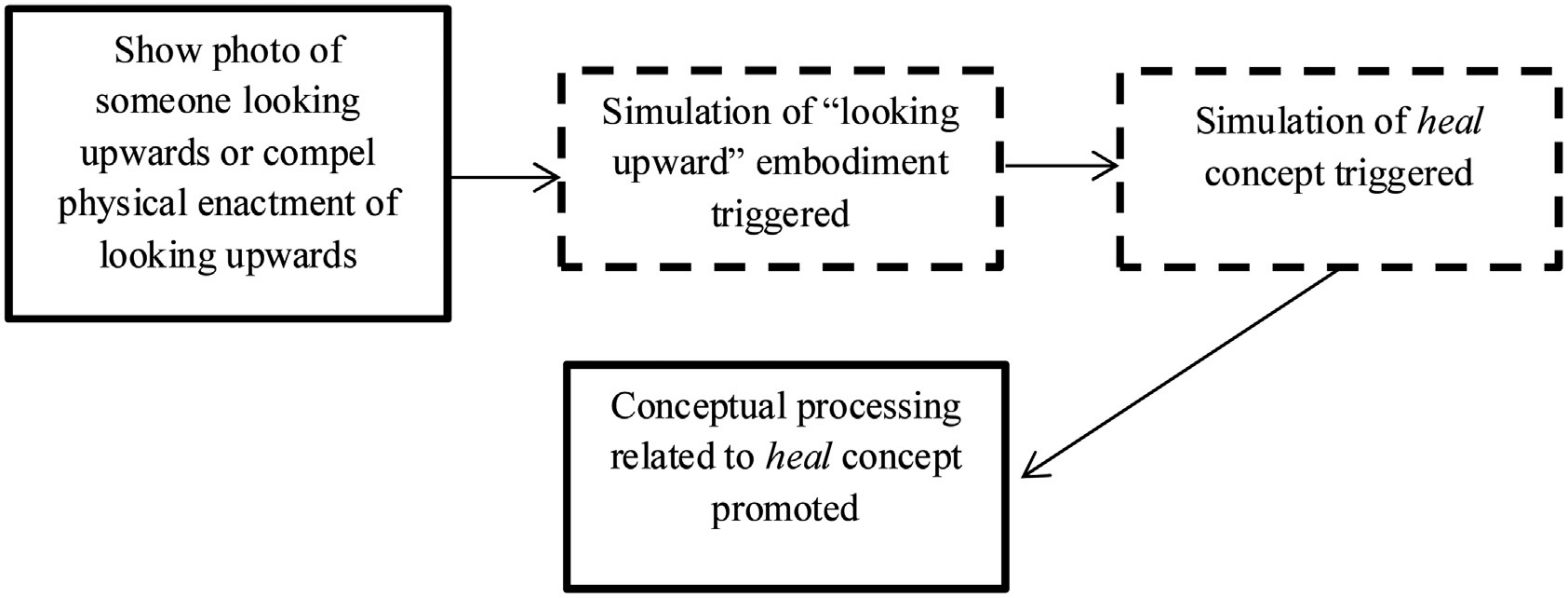
Theorised course of events, as deduced from PSS theory (solid-lined boxes signify observable events, dotted-line boxes signify putative events).

Following PSS theory, we predicted that simulation activated by an image of gazing upward, compared to gazing downward, would promote conceptual processing of *heal* (see Fig 1). Conceptual processing of the *heal* concept was operationalised in a lexical decision making task, using the common assumption that speed of processing and accuracy are the most reliable measures of cognition [33]. For the present study, speed of processing was the major outcome variable while accuracy was used to ensure that participants understood the words in the task.

**Fig 2:**
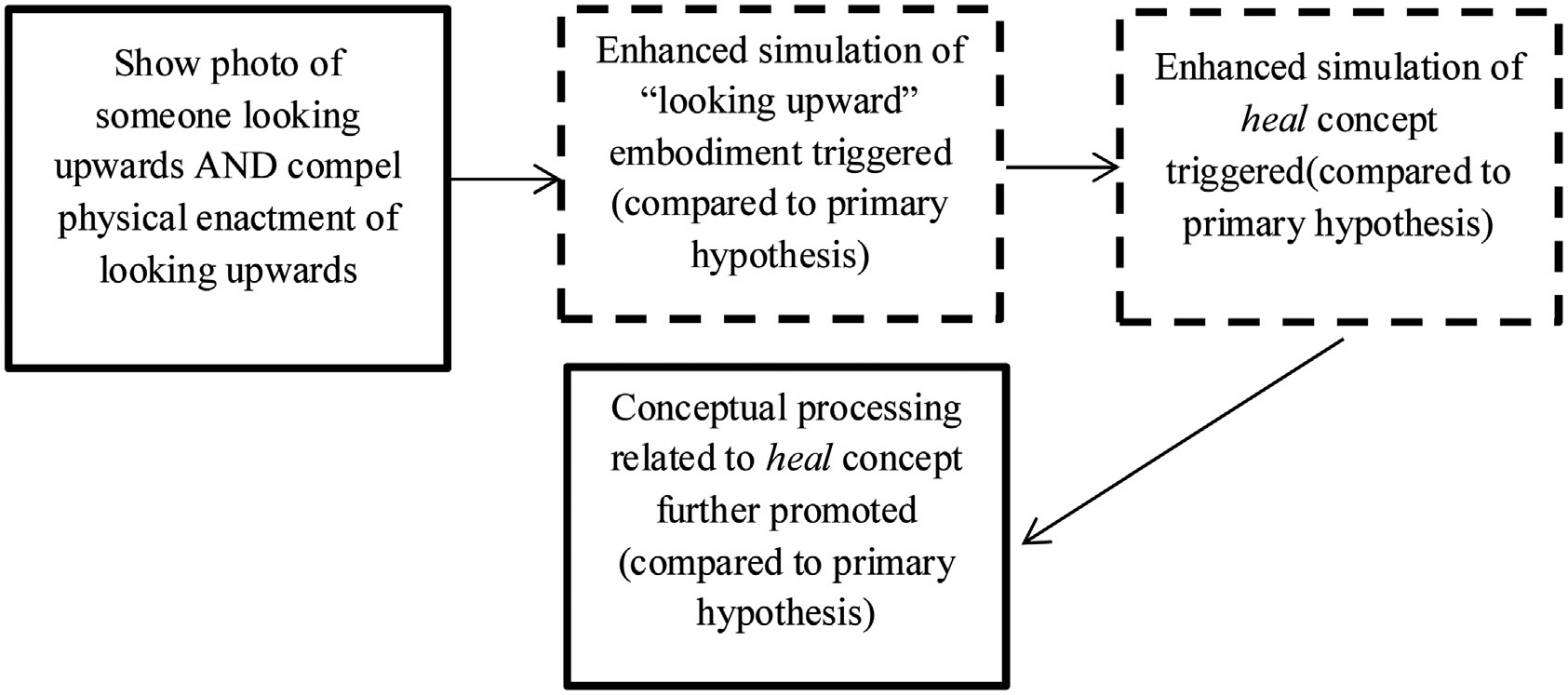
Theorised course of events, as deduced from PSS theory (solid-lined boxes signify observable events; dotted-line boxes signify putative events).

Furthermore, we aimed to explore the proposition that prime “strength” would influence conceptual processing. Specifically, we explored whether congruent motor and visual primes of looking upward, compared to looking downward (high strength), would promote conceptual processing to a greater degree than using a visual prime alone (low strength; see Fig 2).

## Method

### Participants

Participants were 60 university students and staff (30 female; mean age = 29.38 years). “Direction” was a fixed, within subjects factor and “Strength” was a fixed, between subjects factor in the analysis, so participants were randomly allocated into two groups: a low strength group, and a high strength group. Testing sessions took approximately 60 minutes, and participants were reimbursed $20 for participation. Sample size is consistent with those in previous studies of embodied conceptual knowledge (e.g. [34, 35]) and was established before data collection began.

### Procedure

#### Materials

Lexical Stimuli. The lexical stimuli were 36 words related to *heal* and 36 words unrelated to *heal* (total of 72 trials) which were generated by the investigator from various thesauruses [36–38] and word lists [39–42]. These were validated by 19 people who rated every word’s relationship to the *heal* concept (e.g., if the word describes *heal* or describes how you feel when you are “healed”) on a scale from 1 (*not related at all*) to 10 (*highly related*). The mean rating for the group of words assumed to be related to *heal* was 7.78 (*SD* = 2.61) and for the group of words assumed not related to *heal* was 2.35 (*SD* = 2.21) and that this difference was significant (Wilcoxon signed-rank test *Z* = -21.24, *p* < .001). Words were then matched for a number of variables which might influence reaction times including word length (number of letters; [43]), word frequency/familiarity (via the "WordCount" website, [44, 45]), and their linguistic relationship to the word “heal” (since words should only differ between groups on conceptual relationship to heal, not linguistic relationship. Latent Semantic Analysis (LSA) was used to determine linguistic relationships, for a review of LSAand the website used to perform LSA analysis see [46, 47]). They were then spilt into four groups, two 18 word “heal concept” groups and two 18 word “non-heal concept” groups (see S1 Table). These were used to counterbalance the presentation of the words across the up and down conditions. The sets of “heal concept” and “non-heal concept” related words presented in each direction condition were counterbalanced between participants. All words related to *heal* were positively valenced according to the thesaurus’ and word lists used to generate words related to *heal* (words unrelated to *heal* were positively valenced (matched) to reduce the potential confound of valence).

**Fig 3:**
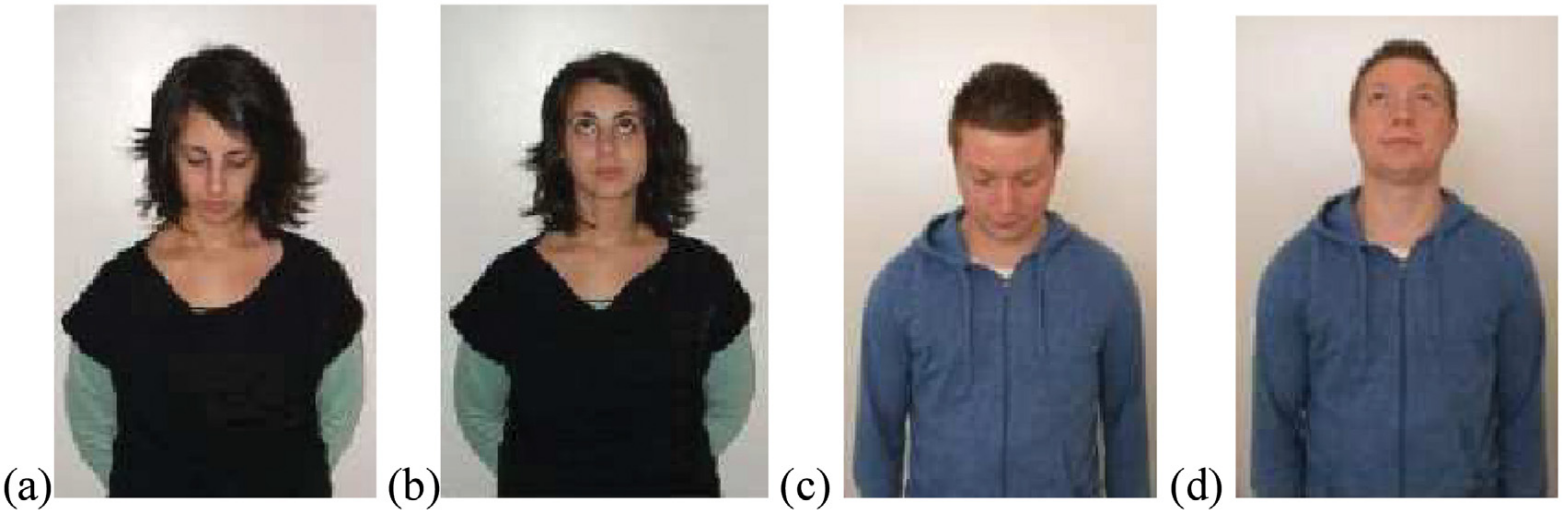
The four photographic visual stimuli used as primes in Study 1.

Visual Stimuli. The visual stimulus was one of two photographs, either a person looking up or looking down (see Fig 3). Visual primes were designed to trigger simulation of the participant performing the action (since embodiments of healing are primarily grounded in experiences of the self), thus it was thought to increase the possibility of simulation if the person in the photograph to be as similar to the participant as possible. Since it was not feasible to match all demographic variables to the participant (e.g., race etc.), gender was matched between participant and photograph (gender congruent photographs used). Gender matching has been considered important in one embodied synchrony study [48].

#### Study protocol

**Fig 4:**
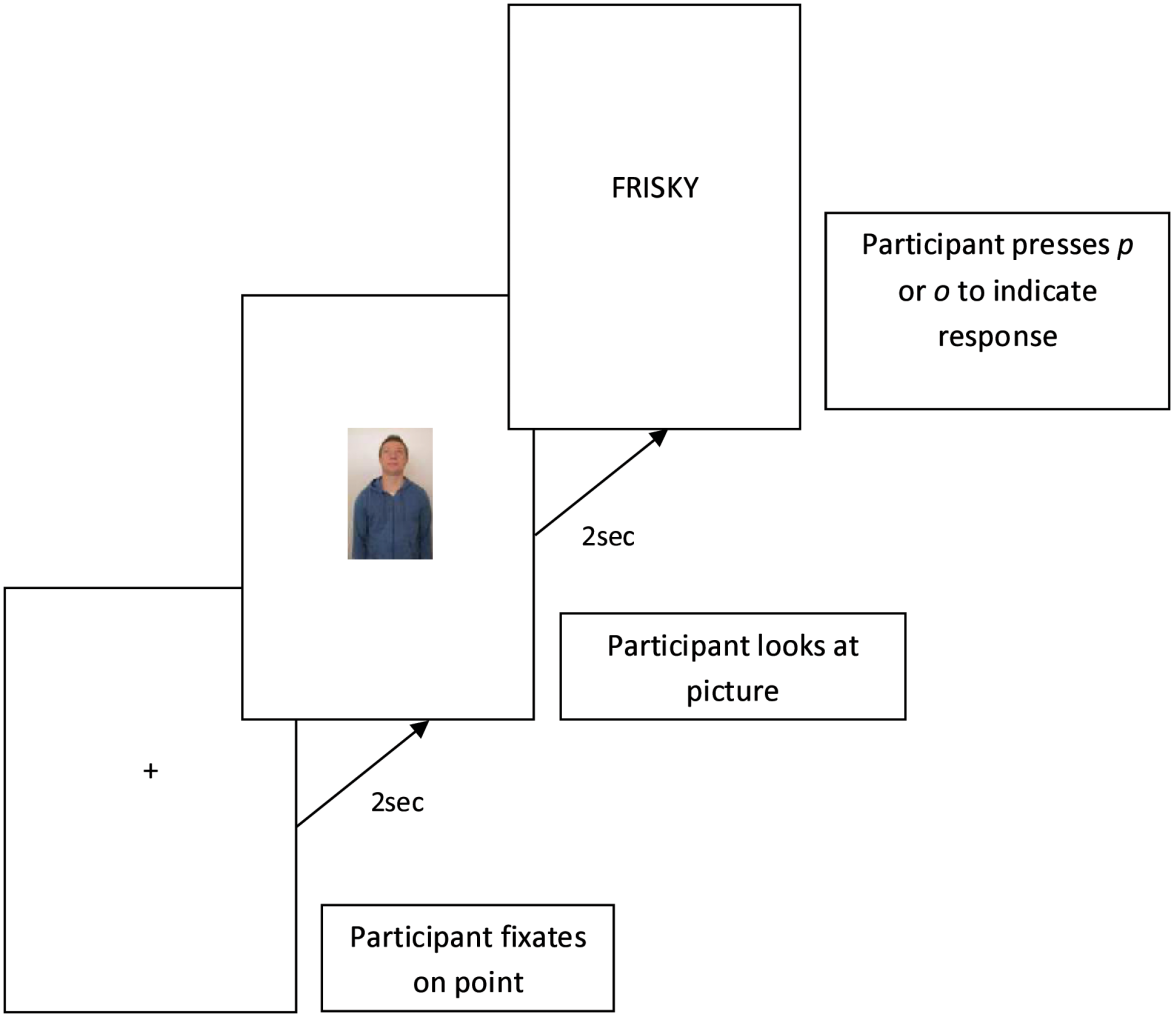
Example of single “up” trial for the low strength group.

**Fig 5:**
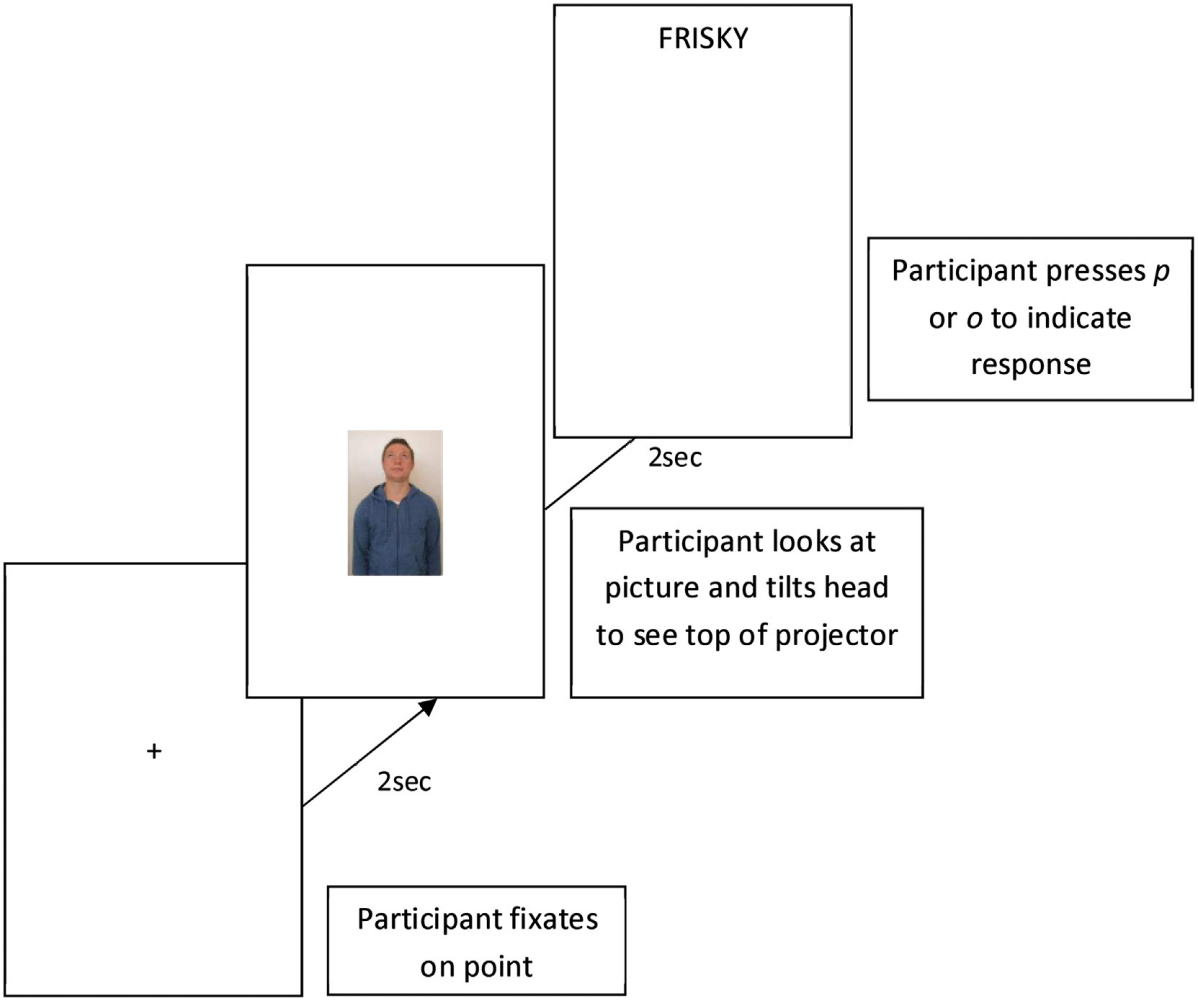
Example of single “up” trial for the high strength group.

Participants were tested individually in a laboratory at Swinburne University of Technology, in Melbourne, Australia. Project was approved by Swinburne University Head Research Ethics Committee (HREC #: 2010/026) and the individuals in Figs 3, 4 and 5 have given written informed consent (as outlined in PLOS consent form) to publish their images. Participants were tested individually in a laboratory at Swinburne University of Technology, in Melbourne, Australia. After providing both written and oral consent, participants were provided with a wireless computer keyboard for making responses and seated in front of a blank white wall onto which the priming task was projected. The distance between the participant and the wall was 1.5m and the projection was 2m x 1m, which placed the vertical centre of the projection approximately at the eye line of the participant (adjusted for their height).

The priming task differed between groups. In the low strength group, a fixation point (+) appeared in the middle of the projection for two seconds after which one of the visual prime stimuli was displayed for two seconds. This was followed by the lexical target, which was displayed in the middle of the projection until the participant responded. Participants were asked to look at the fixation point and then at the visual prime which followed. They were then asked to respond to the following lexical target “as quickly and accurately as possible” by pressing *p* if the word was related to the heal concept or *o* if it was not (*q* and *w* keys were used for left handed participants). After responding, participants were asked to once again look at the fixation point, ready for the next trial. Fig 4 shows a schematic representation of a trial for the low strength group.

For the high strength group, following the visual prime participants were asked to tilt their head either up or down (to focus on the top or bottom of the projection) according to the direction in which the person in the visual stimulus was looking. The lexical target was displayed in the corresponding position until the participant responded. After responding, participants were asked to tilt their head back to the centre of the projection to the fixation point, ready for the next trial. Fig 5 shows a schematic representation of a trial for the high strength.

Participants completed task without the investigator present in the room. Before the experimental stage, participants were asked to complete a practice stage which used the *anger* concept (and words either related or not related to the *anger* concept) in place of the *heal* concept. This stage was designed accustom participants to “concept-related” and “non-concept related” words, response keys, and to the head movements required in the high strength group. The practice stage consisted of 20 trials (10 “anger concept” words and 10 “non-anger concept” words). The investigator stayed in the room for the practice task in order to provide guidance if required. The background colour of the projection was white and words appeared in black 80pt Ariel font (projected size). The protocol was developed using the Inquisit software package (Millisecond Software, Seattle, WA, USA).

## Results

Prior to analysis the data were screened and ANOVA assumptions were tested and met. One participant was found to have performed below the predetermined cut-off point (based on school level language comprehension cut-off percentage; see [49–52]) of 70% correct answers (66% correct answers) and one participant was a univariate outlier in both direction conditions. These were excluded from the analysis, leaving 58 participants (Low strength *n* = 29, High strength *n* = 29). Chi-square tests indicated no significant differences in sex, primary language or background between the two groups (see S2 Table), and independent samples t-tests indicated no significant differences in age or affect between the two groups (see S3 Table).

A 2 (Direction) by 2 (Strength) mixed-design ANOVA was used to test the primary hypothesis and the exploratory research question. Response latency means for up and down conditions for each strength group are displayed in Table 1 (along with dispersion index and proportion correct). The analysis revealed a significant main effect of Direction (*F*(1,56) = 8.37, *p* < .01, partial *η²* = .130) and no differences between groups in dispersion (*F*(1,56) = 2.75, *p* = .10) or proportion of correct answers (*F*(1,56) = .49, *p* = .49). As expected, response latencies for up trials were significantly shorter than those for down trials. Thus, the results were consistent with the hypothesis that priming looking upward promoted faster decision making related to the *heal* concept compared to priming looking downward.

**Table 1:** Response Latency Means for Up and Down Conditions between Strength Groups.

		Direction		
Strength		Up	Down	Total
**Low**	**Mean**	1119.46 [1005.44, 1233.48]	1172.548 [1040.93, 1304.04]	1145.97 [1024.83, 1267.12]
	**Dispersion**	18.43 [16.42, 20.43]	18.99 [16.76, 21.23]	18.71 [16.64, 20.78]
	**Proportion correct**	.71 [.70, .73]	.70 [.69, .71]	.71 [.70, .72]
**High**	**Mean**	1211.34 [1097.31, 1325.36]	1247.61 [1116.06, 1379.17]	1229.65 [1108.33, 1350.62]
	**Dispersion**	20.13 [18.13, 22.14]	20.67 [18.43, 22.90]	20.40 [18.33, 22.47]
	**Proportion correct**	.70 [.69, .71]	.71 [.69, .72]	.70 [.69, .71]
**Total**	**Mean**	1165.40 [1084.77, 1246.02]	1210.05 [1117.03, 1303.07]	
	**Dispersion**	19.28 [17.86, 20.70]	19.83 [18.25, 21.41]	
	**Proportion correct**	.71 [.70, .72]	.70 [.69, .71]	

*N* = 58 (29 per strength group)

*Note*:*-*All latency values in milliseconds

-Dispersion index calculated as SQRT of standard deviation

(Scheffe’s method; [53])

-Proportion correct calculated as arcsin(sqrt(X/n) [54]

-The values in square brackets are 95% confidence intervals.

No significant interaction between Direction and Strength factors was found (*F*(1,56) = .29, *p* = .59). Results also revealed an unpredicted, nonsignificant difference suggesting shorter response latencies for the low strength group compared to the high strength group across direction conditions (*F*(1,56) = 0.95, *p* = 0.33). Thus, the results were not consistent with the idea that congruent motor and visual primes of looking upward, compared to looking downward, would promote conceptual processing to a greater degree than using a visual prime alone.

## Discussion

### Primary hypothesis: Heal grounded in looking upward

The present study found that priming looking upward promoted faster decision making related to the *heal* concept compared with priming of looking downward. This was the first study to examine embodiment of the *heal* concept. The present study suggests that looking upward influences conceptual processing related to the *heal* concept, thereby adding to the literature of demonstration studies suggesting that putative embodiments can influence conceptual processing (prominent examples include [6, 27, 34, 35, 55, 56]).

### Exploratory question: Does combining primes enhance simulation?

This was also the first study to examine the influence of combined primes on the putative process of simulation. Analyses did not find the predicted strength effect of combining primes on decision making related to the *heal* concept. The finding is most likely due to a methodological limitation of the study in which the high strength group had to perform the extra step of tilting their head up or down, which did not have to be performed by the low strength group. This step may have been the cause of the delayed response times seen in the high strength group compared to the low strength group. The strength effect could have been better examined by including a group which only physically tilted their head upward/downward without viewing the image, so that the independent “low strength” effects of visual and motor primes could have been separated from the “high strength” combined effect.

### Caveats of heal as embodied in the up bodily state

A number of caveats regarding our interpretation of this findings as evidence of embodiment of the *heal* concept in looking upward should be noted. Firstly, it may have been that *heal* is linked to “up” because of its innate positive valence and not because of its experiential underpinning by the up bodily state. However, studies examining the relationship between *God* and “up” [56] and *power* and *“*up” [6] have found that the relationship between the respective concepts and “up” is separate from valence. Therefore, it is acknowledged that positive valence may account for *some* of the relationship found between looking upward and the *heal* concept.

Second, it is important to consider whether “looking downward” plays a role in interfering with access to the *heal* concept due to its embodiment of ill health or whether it interferes due to its inverse relationship with “looking upward”. There has been little empirical work conducted on looking downward in relation to any type of illness concept. However, one study found that higher depressive symptoms were associated with a bias towards lower (down) spatial attention targets compared to higher spatial targets [25], suggesting that looking downward may be an illness embodiment in its own right.

### Limitations

A number of methodological and theoretical limitations of the present study should be noted. First, the study lacked a *direction* control condition (e.g., photograph of person looking at participants, as opposed to up/down) so strong conclusions could not be drawn regarding whether simulation of looking upward promoted processing related to the *heal* concept or whether simulation of looking downward interfered with processing. Second, the study design did not allow for illumination of the independent effects of visual and motor primes since there was no group which *only* performed a physical head tilt. Finally, manipulation checks were not administered, so if participants had seen through the cover story and linked looking upward to the heal concept, they may have non-consciously exhibited a bias towards the expected direction of the findings.

### Implications

The present study has potential practical implications for health in a number of ways. If enhanced health cognitions improve perceived health, and looking upward promotes cognitive processing related to health, then people should be encouraged to actively posture their body to look upwards to improve their perception of health. Health practitioners might also use such posturing as a strategy for their patients to improve perceived healing from physical or psychological pain. There has been some evidence to suggest that bodily manipulation is associated with psychological health [57–59], and motor manipulation has been proposed as treatments for depression [60].

Furthermore, health promotion literature might be more effective if placed in higher spatial areas requiring people to look up towards it. Along the same lines, verbal information about health may be more effective if delivered from a higher plane, thus adding to the increasing literature around embodiment in health settings [61–63].

Finally, the present study also opens up new and interesting questions regarding the design of studies examining embodied cognition including the effect of combining primes and the use of photographic stimuli. Finally, our findings encourage future research to examine whether looking upward might not only promote processing related to the *heal* concept but also real-world physical or psychological healing.

### Conclusion

The findings of the present study suggest that, as predicted by a PSS account of conceptual knowledge, simulation of looking upward promotes processing related to the *heal* concept. Future research including appropriate control groups is required to clarify whether combining primes further promotes conceptual processing or whether there may be a ceiling effect reached such that there might be no further promotion of processing and whether looking upward might also improve real world healing.

## Supporting Information

S1 File (SAV)

S1 Table Heal and Non-Heal concept word groups and LSA analysis results. (DOCX)

S2 Table Pearson’s Chi Square statistics for sex and primary language for low strength and high strength groups. (DOCX)

S3 Table Group means and t-test statistics for age and affect for low strength and high strength groups. (DOCX)
