# Supplementary material for: Look Up for Healing: Embodiment of the Heal Concept in Looking Upward
Source: PLoS One. 2015 Jul 10;10(7):e0132427. doi: 10.1371/journal.pone.0132427 (PMC4498772; doi:10.1371/journal.pone.0132427)
Supplement: S1 Table — (DOCX) [file pone.0132427.s002.docx]

| **Heal 1** | **LSA value (healed)** | **Heal 2** | **LSA value (healed)** | **Non-Heal 1** | **LSA value (healed)** | **Non-Heal 2** | **LSA value (healed)** |
| --- | --- | --- | --- | --- | --- | --- | --- |
| well | 0.21 | rejuvenated | -0.01 | frisky | 0.07 | considerate | 0.04 |
| revived | 0.07 | remedied | 0 | clever | 0.16 | provocative | 0.04 |
| alright | -0.04 | renewed | 0.06 | loved | 0.21 | captivating | 0.22 |
| better | 0.15 | restored | 0.08 | appreciated | 0.23 | privileged | 0.03 |
| healthy | 0.06 | good | 0.13 | honoured | 0.07 | attractive | 0.05 |
| cured | 0.23 | uplifted | 0.04 | adored | 0.07 | intelligent | 0.09 |
| risen | 0.08 | elated | 0.17 | idolised | -0.04 | sophisticated | 0.06 |
| lifted | 0.18 | improved | 0.11 | engaged | 0.09 | charming | 0.07 |
| refreshed | 0.06 | alive | 0.19 | smart | 0.11 | humorous | 0.1 |
| thankful | 0.11 | joyful | 0.1 | prosperous | 0.08 | respected | 0.18 |
| regenerated | 0.03 | delighted | 0.13 | passionate | 0.16 | sexy | 0.12 |
| rehabilitated | 0.19 | happy | 0.1 | surprised | 0.17 | trusted | 0.08 |
| optimistic | 0.05 | fit | 0.17 | beautiful | 0.15 | honest | 0.17 |
| revitalised | 0.02 | energised | 0 | seductive | 0.03 | proud | 0.22 |
| relieved | 0.19 | lively | 0.15 | affectionate | 0.1 | unselfish | 0.07 |
| strengthened | 0.1 | aroused | 0.06 | fascinated | 0.19 | cool | 0.07 |
| stimulated | 0.05 | enthusiastic | 0.08 | intrigued | 0.04 | friendly | 0.12 |
| empowered | -0.01 | pleased | 0.19 | impulsive | 0.04 | generous | 0.19 |
