# Supplementary material for: Look Up for Healing: Embodiment of the Heal Concept in Looking Upward
Source: PLoS One. 2015 Jul 10;10(7):e0132427. doi: 10.1371/journal.pone.0132427 (PMC4498772; doi:10.1371/journal.pone.0132427)
Supplement: S2 Table — (DOCX) [file pone.0132427.s003.docx]

Pearson’s Chi Square statistics were conducted to compare any potentially confounding differences in sex, primary language and background. As displayed in S2 Table, there were no significant differences in sex (*X²* (1) = .28, *p* = .60)*,* primary language (*X²* (2) = 2.99, *p* = .22) or background (*X²* (2) = 0, *p* = 1) between the two groups.

**S2 Table. Pearson’s Chi Square statistics for sex and primary language for low strength and high strength groups**

|  | | | **Group** | | | |
| --- | --- | --- | --- | --- | --- | --- |
|  | | | **Low strength** | | **High strength** | |
| **Variable** | **Values** | ***N*** | **Frequency** | **%** | **Frequency** | **%** |
| **Sex** | **Male** | 30 | 14 (15) | 24.14 | 16 (15) | 27.59 |
|  | **Female** | 28 | 15 (14) | 25.86 | 13 (14) | 22.41 |
| **Primary Language** | **English** | 33 | 18 (16.5) | 31.03 | 15 (16.5) | 25.86 |
|  | **ESL** | 19 | 10 (9.5) | 17.24 | 9 (9.5) | 15.52 |
|  | **Bilingual** | 6 | 1 (3) | 1.72 | 5 (3) | 8.62 |
| **Background** | **Western** | 30 | 15 (15) | 25.86 | 15 (15) | 25.86 |
|  | **Eastern** | 24 | 12 (12) | 20.69 | 12 (12) | 20.69 |
|  | **Both** | 4 | 2 (2) | 3.45 | 2 (2) | 3.45 |

*N* = 58 (29 per strength group)

ESL = English second language

^a^ The Chi Square assumption of “expected cell frequency” is violated where 16.67% of cells have expected frequencies <5.

NB. Expected cell frequencies are shown in parentheses
