# Supplementary material for: Look Up for Healing: Embodiment of the Heal Concept in Looking Upward
Source: PLoS One. 2015 Jul 10;10(7):e0132427. doi: 10.1371/journal.pone.0132427 (PMC4498772; doi:10.1371/journal.pone.0132427)
Supplement: S3 Table — (DOCX) [file pone.0132427.s004.docx]

A series of independent samples t-tests were conducted to compare any potentially confounding differences in age and affect between the two groups. As displayed in S3 Table, there were no significant differences in age or affect between the two groups.

**S3 Table. Group means and t-test statistics for age and affect for low strength and high strength groups**

| **Variable** | **Values** | **Low strength**  **Mean (*SD*)** | **High strength**  **Mean (*SD*)** | ***t*** | ***df*** | ***p*** |
| --- | --- | --- | --- | --- | --- | --- |
| **Age** |  | 30.21 (12.56) | 28.75 (10.71) | 0.469 | 54 | .641 |
| **Affect** | **PA** | 3.34 (.66) | 3.03 (.71) | 1.732 | 56 | .089 |
|  | **NA** | 1.34 (.26) | 1.25 (.24) | 0.701 | 56 | .486 |

PA = Positive Affect

NA = Negative Affect

*N_age_* = 56 (28 per strength group - 2 missing values, 2 excluded in initial data

screen)

*N_PA_* = 58 (29 per strength group, 2 excluded in initial data screen)

*N_NA_* = 58 (29 per strength group, 2 excluded in initial data screen)
